# Transferrin improved the generation of cardiomyocyte from human pluripotent stem cells for myocardial infarction repair

**DOI:** 10.1007/s10735-020-09926-0

**Published:** 2020-11-11

**Authors:** Fengzhi Zhang, Hui Qiu, Xiaohui Dong, Chunlan Wang, Jie Na, Jin Zhou, Changyong Wang

**Affiliations:** 1 Department of Neural Engineering and Biological Interdisciplinary Studies, Institute of Military Cognition and Brain Sciences, Academy of Military Medical Sciences, Academy of Military Sciences, Beijing, China; 2 School of Medicine, Tsinghua University, Beijing, China

**Keywords:** hPSC, Cardiomyocyte, Chemically defined, Transferrin, Myocardial infarction

## Abstract

Human pluripotent stem cell (hPSC)-derived cardiomyocytes (CMs) hold great promise for the repair of the injured heart, but optimal cell production in a fully chemically defined and cost-effective system is essential for the efficacy and safety of cell transplantation therapies. In this study, we provided a simple and efficient strategy for cardiac differentiation from hPSCs and performed functional evaluation in a rat model of myocardial infarction. Using a chemically defined medium including four components, recombinant human albumin, ascorbic acid, human transferrin, and RPMI 1640, we developed a manageable and cost-effective protocol for robust generation of CMs from hPSCs. Interestingly, the addition of transferrin helped hPSCs to transit from TeSR-E8 medium to the simple cardiac differentiation medium and successfully initiated mesoderm differentiation without significant cell death. The CM generation efficiency was up to 85% based on cTnT expression. We performed transcriptome profiling from differentiation day 0 to 35, and characterized interesting dynamic change of cardiac genes. CMs derived from transferrin-supplemented simple medium have similar transcriptome and the maturation level compared to those generated in B27 minus insulin medium as well as their in vivo counterparts. Importantly, after transplantation, hPSC-derived CMs survived in the infarcted rat heart, significantly improved the physiological function and reduced fibrosis. Our study offers an easy-to-use and cost-effective method for cardiac differentiation and facilitates the translational application of hPSC-derived CMs for heart repair.

**Electronic supplementary material:**

The online version of this article (10.1007/s10735-020-09926-0) contains supplementary material, which is available to authorized users.

## Introduction

Adult mammalian hearts have a limited regenerative capacity, consequently, loss of cardiomyocytes is a leading cause of heart failure (Laflamme and Murry 2011; Weinberger and Eschenhagen 2020). Cardiomyocyte transplantation is a straight forward approach for cardiac repair and human pluripotent stem cells (hPSCs) offer an attractive cell source for cardiomyocyte generation. hPSCs, including human embryonic stem cells (hESCs) and human induced pluripotent stem cells (hiPSCs) (Thomson et al. 1998; Yu et al. 2007), can indefinitely proliferate and efficiently differentiate into functional cardiomyocytes in large numbers. In recent years, accumulating evidence reveals that monolayer-based cardiac differentiation of hPSCs is easier to manipulate and consistently produce a higher yield compared to embryoid body (EB)-based methods (Batalov and Feinberg 2015). Appropriate temporal modulation of Wnt signaling alone using small molecule inhibitors is sufficient for efficient cardiomyocyte differentiation. Meanwhile, Lian et al. employed a completely defined and growth factor-free medium for scalable production of cardiomyocytes from hPSCs, such as the basal medium RPMI 1640 supplemented with B27 minus insulin (B27-ins) for cardiac differentiation and B27 for subsequent cardiomyocyte maintenance (Lian et al. 2012, 2013b).

Of note, B27 is a complex mix containing 21 components, many of which are derived from animal origin. There are apparent drawbacks when introducing complex animal-derived factors to human cardiomyocyte production, such as impeding elucidation of underlying mechanism, influencing reproducibility of differentiation, as well as increasing health risks for downstream application. In 2014, Burridge et al. published a chemically defined and low-cost cardiac differentiation medium (termed CDM3) containing just three components, RPMI 1640, recombinant albumin, and ascorbic acid, which is by far the simplest medium formula (Burridge et al. 2014). However, we observed that the sudden transition from TeSR-E8 medium which contains high levels of insulin and transferrin to CDM3 frequently led to a marked increase of cell death, and consequently, reduced the final CM yield. The extent of cell death varies depending on the condition of the starting hPSCs and the person carrying out the experiment. We reasoned that the addition of the transferrin might be beneficial for cell survival and smooth transition from TeSR-E8 medium to simple cardiac differentiation medium like CDM3. As expected, the transferrin supplement enabled hPSCs to go through cardiac differentiation without much cell loss. The efficiency of cTnT^+^ CMs formation reached up to 85%. Importantly, upon transplantation into a rat model of myocardial infarction (MI), hPSC-derived CMs improved remuscularization of the infarcted area and the function of the heart.

## Materials and methods

### hPSC culture

H1, H9 hESCs (WiCell Institute), HUES8 hESCs (Harvard Stem Cell Institute), and human induced pluripotent stem cells (CD34-iPSCs Zhang et al. 2017; Duan et al. 2018), provided by Dr. J. Na, Tsinghua University) were routinely maintained on Matrigel (BD Biosciences)—coated plates (Corning) in TeSR-E8 medium (STEMCELL Technologies). hPSCs were passaged every 4 days using Accutase (Merck) and 10 μM ROCK inhibitor Y27632 (Selleck) was added for the first 24 h after passaging. Mycoplasma testing was performed for all cell cultures using a MycoAlert Kit (Lonza).

### Cardiac differentiation of hPSCs

Both hESCs and hiPSCs maintained in TeSR-E8 medium were digested into single cells by Accutase (Millipore) and plated onto Matrigel-coated culture dishes at a density of 1–2 × 10^4^ cells/cm^2^ (adjust the seeding density to reach ~ 90% confluence in 4 days according to the growth rate of different hPSC lines) in TeSR-E8 medium with 10 μM Y27632. After 24 h, Y27632 was withdrawn from the medium and cells were cultured in TeSR-E8 medium for 3 days. At day 0, cells were treated with 4 μM CHIR99021 (Selleck) for 2 days in a basic differentiation medium, consisting of RPMI 1640, 0.5 mg/mL recombinant human serum albumin (ORYZOGEN), 0.2 mg/mL Ascorbic Acid 2-Phosphate Magnesium (Sigma), and 5 μg/mL human Apo-Transferrin (Sigma). This basic culture medium, referred to as transferrin-supplemented AA medium, was used for cardiac differentiation and initial maintenance of beating CMs. After 2 days, the medium was changed to the basic differentiation medium supplemented with 5 μM IWP2 (Selleck) for another 2 days. At day 4, the cells were continuously cultured in the basic differentiation medium for another 10 days, which was changed every other day. Contracting cells were seen from day 9 to day 10. From day 14 to day 30, 4 μg/mL insulin (Sigma) was added to the basic differentiation medium, and the medium was changed every other day.

### Flow cytometry analysis

Cells were dissociated into single cells with CardioEasy solution (CELLAPY) and fixed with 4% (wt/vol) paraformaldehyde for 15 min at room temperature, permeabilized with 0.1% (vol/vol) Triton X-100, and processed with PBS with 5% (vol/vol) fetal calf serum and 2.5 mM EDTA. Cells were stained with primary antibodies (cTnT, 1:100, R&D and α-actinin, 1:100, Abcam) for 1 h at room temperature and labeled with DyLight 488-conjugated secondary antibodies (1:100) for 30 min at room temperature. Data were collected on a FACSCaliber flow cytometer (BD Biosciences) and processed using FlowJo 10.4 software (TreeStar).

### Immunostaining analysis

Cells were fixed with 4% paraformaldehyde for 15 min at room temperature, permeabilized with 0.1% (vol/vol) Triton X-100 (Sigma) for 10 min at room temperature, and blocked in 10% (vol/vol) normal goat serum (Origene), and then incubated with primary antibodies (Supplementary Table 1) overnight at 4 °C and secondary antibodies (Supplementary Table 2) for 1 h at room temperature. Nuclei were stained with DAPI (Sigma). Nikon Ti-U fluorescence microscope and A-1 confocal microscope was used for image capture.

### Calcium transient detection and analysis

10 μM Fluo-4 AM (Thermo) was prepared and added to cells according to standard procedures. Cells were incubated for 10 min at 37 °C and time-lapse fluorescence images were recorded using an Opera Phenix HCS System (PerkinElmer). Data were processed using ImageJ software.

### Bulk RNA-seq and data analysis

RNA samples were harvested at seven time points (day 0, 2, 3, 5, 15, 21, and 35) during cardiac differentiation of hiPSCs. Total RNA was extracted using Trizol (Invitrogen), and then 100 ng of RNA of each sample was reverse-transcribed using Superscript III (TransGen). cDNA libraries were prepared using Smart-seq2 protocol and RNA sequencing was performed using Illumina HiSeq X Ten system.

Quality of raw sequencing data was inspected using FastQC (v0.11.8), and then adapter sequences were trimmed using TrimGalore (v0.6.4). The resulting clean data were mapped to the human reference genome (GRCh38) using STAR (2.7.3a) with—quantMode GeneCounts option to output read count tables. Count tables of all samples were combined and differential gene expression analysis was performed using DESeq2 (v1.24.0). Data are publicly available at the National Center for Biotechnology Information with Gene Expression Omnibus (GEO), accession number GSE154294.

### Reverse transcription and Q-PCR analysis

1 μg RNA of each sample was reverse-transcribed with 5 × All-In-One RT MasterMix (abm). Q-PCR reactions were performed using GoTaq qPCR Master Mix (Promega) in a CFX96 Real-Time System (Bio-Rad) and results were analyzed with the Bio-Rad CFX Manager program. The sample input was normalized against the Ct (Critical threshold) value of *GAPDH*. Primer sequences are listed in the Supplementary Table 3.

### MI model and cell transplantation

All animal procedures in this study were approved by the Institutional Animal Care and Use Committee (IACUC) of the Chinese Academy of Military Medical Science. Acute MI was induced in male Sprague–Dawley rats (250 ± 10 g) using an established protocol (Wang et al. 2020). Briefly, rats were anesthetized by intraperitoneal administration of sodium pentobarbital (30 mg/kg, Sigma) and the left anterior descending coronary artery (LAD) was then ligated with 6–0 Prolene suture. After ten minutes, the infarct region was confirmed by myocardial blanching. Injections were performed along the border zone of the infarcted area at 3 sites (below the left atrium, in the middle portion of the left ventricle, and at the apex) with a total volume of 100 μl using a 28 gauge needle. Rats were randomly divided into 3 groups: Sham group (n = 6), Control group (n = 6), and Cell treat group (n = 6). Cell treat group was given hiPSC-derived CMs (8 × 10^6^) and the detailed protocols were described elsewhere (manuscript submitted), Control group was injected with PBS alone, and Sham group was subjected to thoracotomy and cardiac exposure without coronary ligation.

### Echocardiography and histology analysis

Echocardiography was performed 28 days after transplantation using a Vevo 2100 system (Visualsonic, Toronto, Canada). Using M-mode tracings, the following parameters were measured: left ventricle ejection fraction (LVEF) and left ventricle fractional shortening (LVFS). After functional measurements, hearts were removed and fixed with 4% PFA overnight at 4 °C and paraffin sections were prepared for Masson’s trichrome staining, human cTnT (Abcam) staining, and human CD31 (Abcam) staining according to standard procedures. Images were captured with a slide scanner microscope Axio Scan. Z1 (Carl Zeiss, Gottingen, Germany) and ZEN software (Carl Zeiss). The fibrotic area was measured using ImageJ software.

### Statistical analysis

Quantitative data are presented as mean ± SD. The statistical significance was determined using a Student’s *t*-test (two-tail) for two groups or one-way ANOVA for multiple groups. A value of *p* < 0.05 was considered statistically significant.

## Results

### Transferrin supplement reduced cell death during CM differentiation from hPSCs

**Fig. 1:**
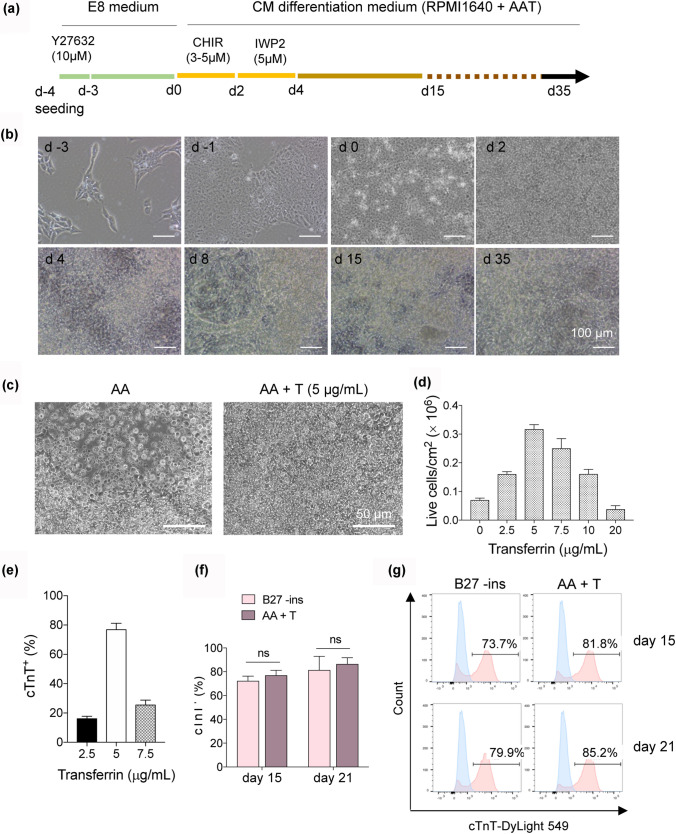
Transferrin supported CM differentiation from hPSCs. **a** A schematic of the optimized protocol of CM differentiation from hPSCs. A, L-ascorbic acid 2-phosphate; A, recombinant human albumin; T, Apo-transferrin. **b** Representative phase contrast images of human CD34-iPSC during transferrin-supported chemically defined differentiation. **c** Representative phase contrast images of human CD34-iPSC on day 2 in AA and AA + T. **d** Yield of live cells on day 2 when varying dose of transferrin. **e** Flow cytometry assessment of expression of cardiac Troponin T (cTnT) on day 15 when varying dose of transferrin. **f** The efficiency of CM differentiation in the indicated medium, measured by flow cytometry for cTnT on day 15 and day 21, respectively. B27-ins, B27 minus insulin. **g** Representative flow cytometric histogram of cTnT^+^ cells generated from the indicated medium on day 15 and day 21, respectively

We noticed frequent cell death during our cardiac differentiation procedure using medium only supplemented with rice-derived recombinant human **a**lbumin and L-ascorbic acid 2-phosphate in RPMI 1640 basal medium (hereafter referred to as AA). The most severe cell loss occurred after change TeSR-E8 medium to AA. TeSR-E8 medium contains high concentrations of insulin and transferrin (Chen et al. 2011). While insulin is omitted from the cardiac differentiation medium due to its inhibitory effect on cardiac mesoderm induction (Freund et al. 2008; Lian et al. 2013b; Wang et al. 2020), the effect of the transferrin is not clear. Here, we added human transferrin to the AA medium and found it significantly reduced cell loss during cardiac differentiation (Fig. 1a–d). The differentiation process is shown in Fig. 1a. hPSCs were maintained in TeSR-E8 medium for 4 days until reaching ~ 90% confluence. At day 0, the medium was changed to RPMI 1640 supplemented with rice-derived recombinant human albumin, L-ascorbic acid 2-phosphate, and human transferrin, and the small molecules CHIR99021 and IWP2 were sequentially added to modulate WNT signaling (Fig. 1a). Using this procedure, many beating areas can be seen in the culture on differentiation day 8 (Fig. 1b and Supplementary Movie 1). The optimal transferrin concentration was 5 μg/mL, which produced the highest yield of cTnT^+^ cardiomyocytes (Fig. 1c-e and Supplementary Fig. 1). By differentiation day 21, the culture contained wide spread spontaneous beating areas. The rhythmic contraction of hPSC-derived CMs was evident based on the frequency of their Ca^2+^ influx (Supplementary Fig. 2 and Supplementary Movie 2).

**Fig. 2:**
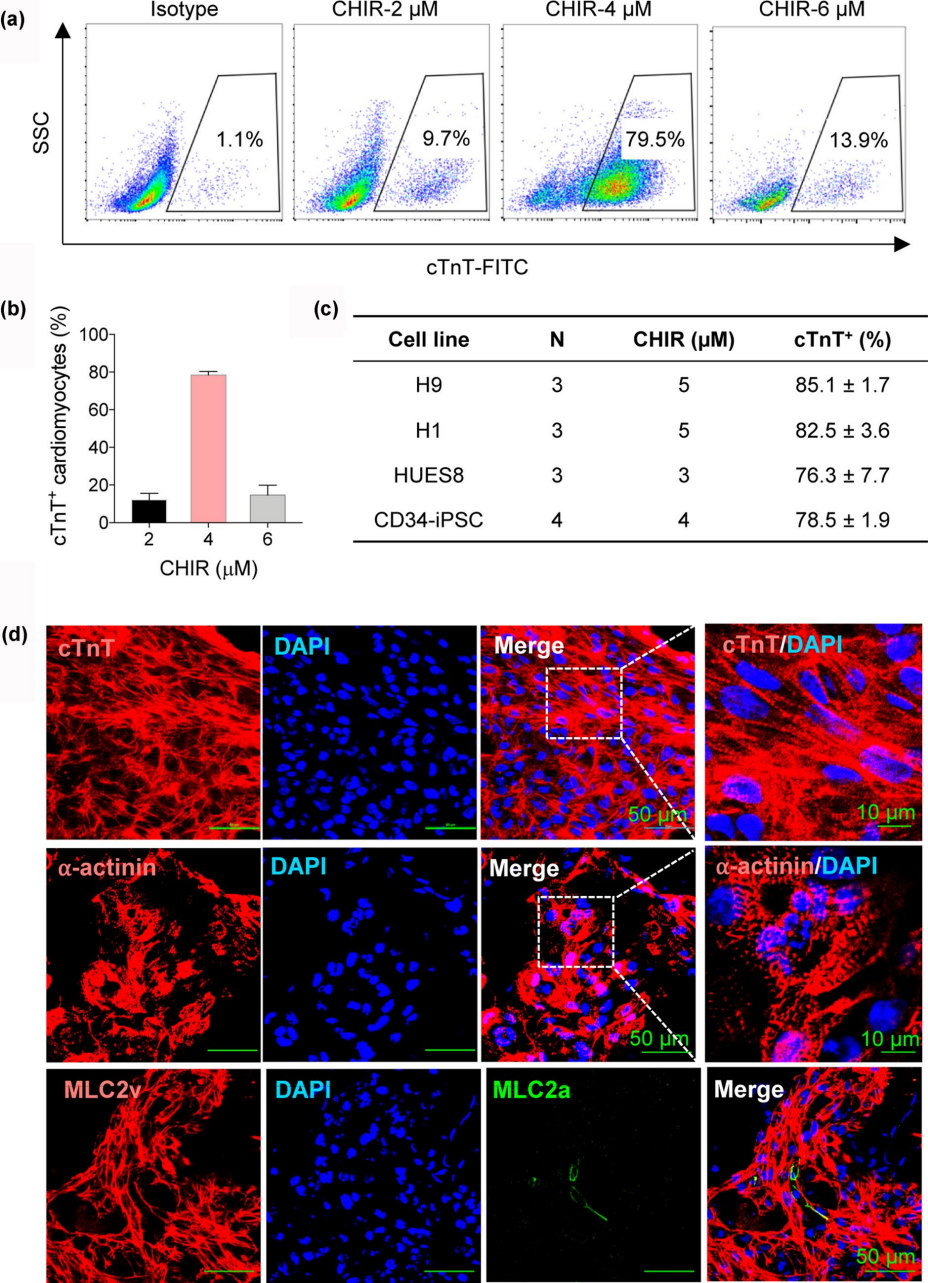
Optimization of CHIR dose for mesoderm induction and characterization of hPSC-derived CMs. **a** Flow cytometric analysis of cTnT^+^ populations induced with different dose of CHIR in the transferrin-supplemented AA medium. **b** Bar graph showing the cardiac differentiation efficiency under the indicated CHIR dose for at least 3 independent replicates. **c** Cardiac differentiation efficiency from various hPSC lines and the optimal treatment dose of CHIR. **d** Immunostaining of day 21 CMs with the indicated antibodies. Scale bars, 50 µm and 10 µm (zoom-in views of the indicated regions)

RPMI 1640 medium supplemented with B27 minus insulin (B27-ins) is often used for cardiac differentiation. Our transferrin-supplemented AA medium (AA + T) generated 81.8% and 85.2% cTnT^+^ cells at differentiated day 15 and 21, respectively. While B27 -ins medium produced 73.7% and 79.9% cTnT^+^ cells at day 15 and day 21 (Fig. 1f and g). Thus, beyond ensuring cell survival, the addition of transferrin can robustly generate cardiomyocytes comparable to the results using B27-ins-supplemented medium.

### Highly efficient generation of CMs by modulating the concentration of GSK3 inhibitor

Appropriate modulation of Wnt signaling pathway is sufficient for efficient cardiac differentiation (Lian et al. 2013a). Here, we first used CHIR99021 (GSK3β inhibitor), then IWP2 (inhibitor of Wnt production-2) to activate and suppress canonical Wnt signaling. CHIR99021 treatment can promote the rapid and efficient conversion of hPSCs into mesoderm, which is a crucial step for a successful cardiac differentiation protocol. Next, we optimized the concentration of CHIR99021 treatment between day 0 and 2 in the transferrin-supplemented AA medium. Flow cytometric analysis showed 4 μM CHIR99021 induced cTnT expression most efficiently in CD34-iPSC line, yielding an average of 78.5% cTnT^+^ CMs (Fig. 2a and b). And more notably, the effect of Wnt signaling pathway activation triggered by CHIR99021 was variable for different cell lines, we performed dose–response assays and found a range of 3–5 μM for several representative cell lines. Wnt signaling inhibition with IWP2 was performed after CHIR99021 from day 2 to 4 and the concentration of 5 μM was optimal for CM generation in all tested hPSC lines. Taken together, optimization of the dose of CHIR99021 during the first 2 days is critical for the efficiency of CM differentiation.

To assess the generality and application of our transferrin-supported cardiac differentiation system, we tested multiple hPSC lines (including 3 hESC lines and 1 hiPSC line). The differentiation efficiency of around 80% cTnT^+^ cells was observed by flow cytometric analysis (Fig. 2c). The resultant CMs were characterized by flow cytometric analysis for cTnT as described above. Besides, we evaluated the differentiated CMs by immunostaining and cross-striated distribution of cTnT and α-actinin indicated well-differentiated sarcomeric structures (Fig. 2d). Furthermore, the two major isoforms of the myosin light chain, including MLC2a and MLC2v, have been used to identify the subtype and maturity of hPSC-derived CMs (Lian et al. 2012; Zhang et al. 2012; Burridge et al. 2014). MLC2v expression is restricted to ventricles and persists into adulthood, whereas MLC2a expression is present in both atrial and ventricular chambers in the developing heart and lost in mature ventricular myocytes. Immunostaining for MLC2a and MLC2v in CMs on differentiated day 21 showed large amounts of MLC2v^+^ cells and very few MLC2a^+^ cells, indicative of a majority of ventricular cardiomyocytes. Thus, the day 21 CMs differentiated in the transferrin-supplemented medium possessed a predominantly ventricular phenotype.

### Gene expression signatures from hPSCs to CMs

**Fig. 3:**
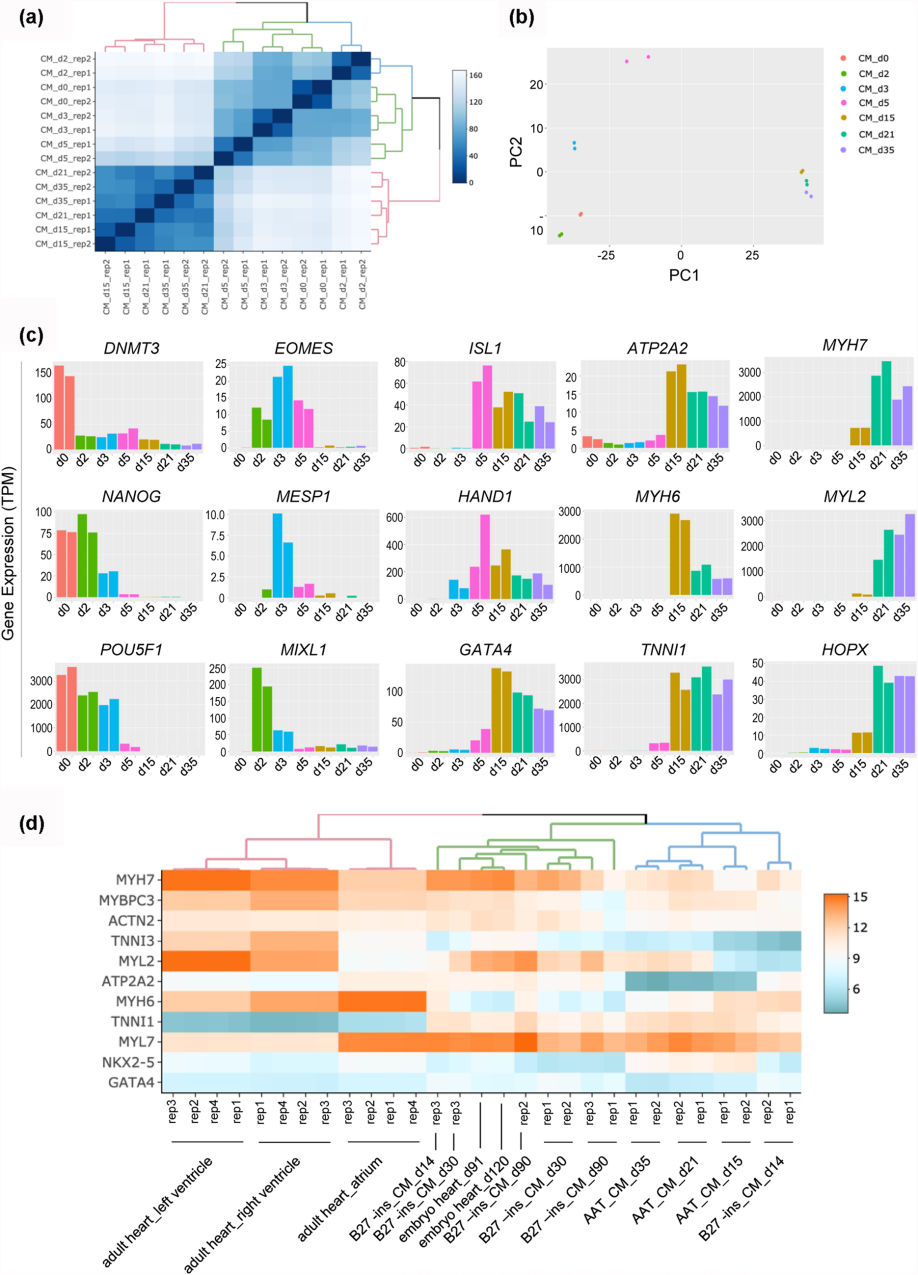
Whole transcriptome analysis of differentiation of hPSCs to CMs. **a** Correlation heatmap for bulk time-course RNA-seq samples. **b** Principal components analysis (PCA) of RNA-seq samples for hiPSCs at differentiated day 0, 2, 3, 5, 15, 21, and 35. **c** Expression level for stage-specific genes during hiPSC-derived cardiac differentiation. pluripotency genes (*DNMT3B*, *NANOG*, and *POU5F1*), mesendoderm genes (*EOMES*, *MESP1*, and *MIXL1*), transcription factors (*ISL1*, *HAND1*, and *GATA4*), calcium handling gene (*ATP2A2*), and sarcomere genes (*MYH6*, *MYH7*, *MYL7*, *MYL2*, and *TNNI1*). **d** Heatmap comparing expression of cardiac genes in hPSC-derived CMs from day 15, 21, and 35 samples relative to samples (d14, d30, and d90 samples) from B27-supplemented medium (J. Churko et al., 2018), as well as human fetal and adult heart samples (ENCODE)

To assess the CMs differentiated in transferrin-supplemented medium and understand the global molecular dynamics of differentiation, we performed bulk RNA-seq analysis of seven time points during cardiac differentiation of hiPSCs, including differentiated day 0, 2, 3, 5, 15, 21, and 35 samples. Spearman correlation coefficients revealed that hiPSCs (day 0) clustered relatively closer to cells at differentiated day 2 and 3, but quite different from cells from day 15 to 35 (Fig. 3a). Principal component analysis (PCA) on the time-course RNA-seq data exhibited a progression in gene expression signatures of hiPSCs undergoing differentiation toward CMs (Fig. 3b). There was a comparatively great difference in gene expression profiling among early differentiation stage points (including day 0, 2, 3, and 5), whereas the CMs at day 15, 21, and 35 were more similar and clustered together.

Temporal expression dynamics of stage-specific genes indicated cardiac fate choice during cardiogenesis (Fig. 3c). In agreement with embryonic development and expression patterns reported in previous cardiac differentiation approaches, the pluripotency makers *DNMT3*, *NANOG*, and *POU5F1* rapidly downregulated followed by transient activation of mesendoderm genes *EOMES*, *MESP1*, and *MIXL1* around day 3. Transcription factors *ISL1*, *HAND1*, and *GATA4* governing cardiac cell specification in the early stage were highly expressed from day 5. The upregulation of calcium handling gene *ATP2A2* and sarcomere genes *MYH6* and *TNNI1* reflected immature cardiomyocytes generated on day 15. High expression of *MYH7*, *MYL2*, and *HOPX* represented further maturation of cardiomyocyte between day 21 and 35. These results were also confirmed by Q-PCR analysis (Supplementary Fig. 4).

**Fig. 4:**
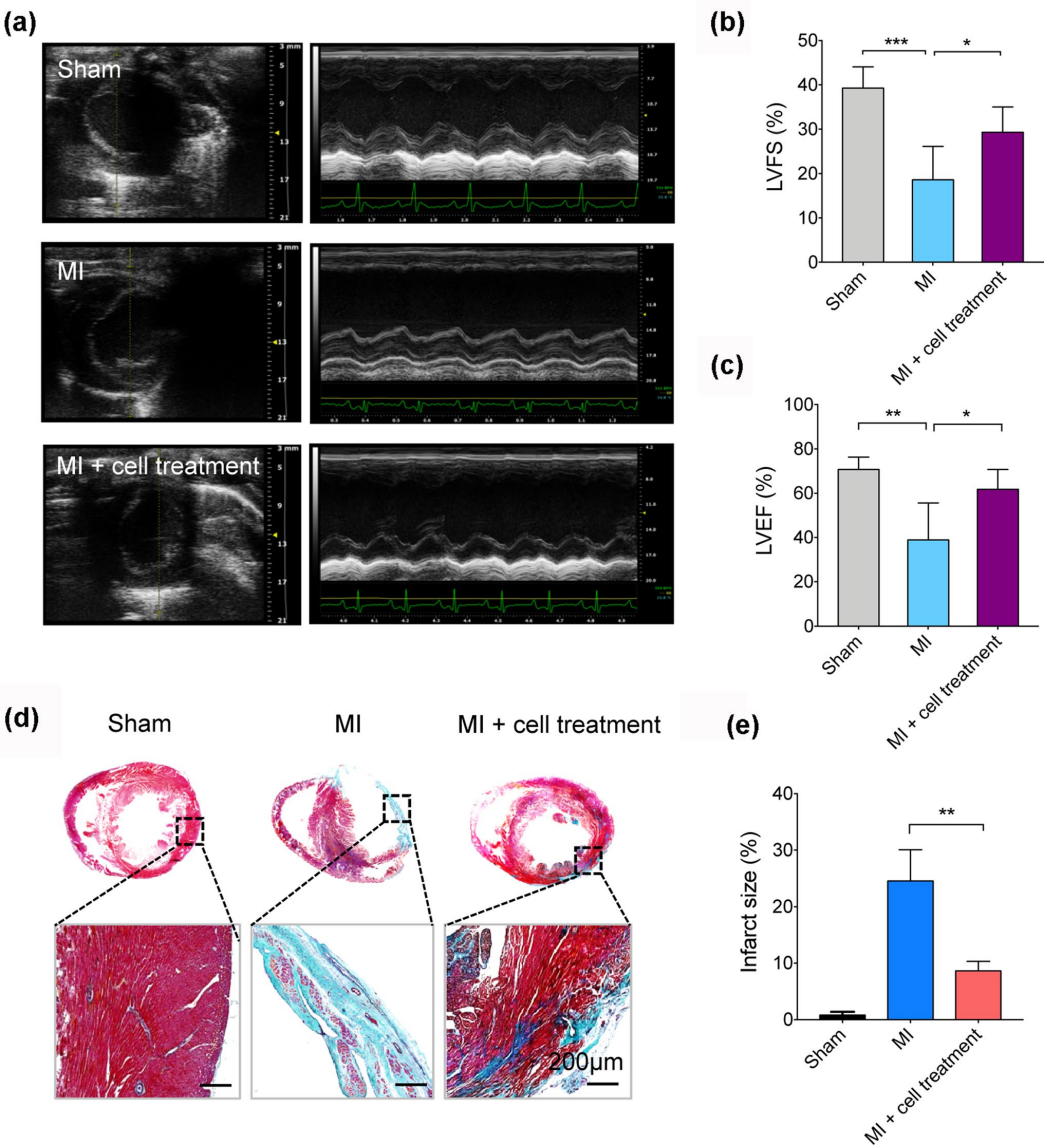
Assessment of cardiac function and infarct size after MI and CM transplantation. **a** Representative M-mode traced echocardiographic images (short axis view) for Sham, MI, and MI + cell treatment groups on day 28 post-transplantation. **b** and **c** Quantitative cardiac function analysis of LVFS and LVEF on day 28 post-transplantation. Data expressed as mean ± SD. n = 6 (Sham and MI groups), n = 8 (MI + cell treatment group). *p < 0.05, **p < 0.005. **d** Representative images of rat heart sections with Masson’s trichrome staining on day 28 post-implantation. Scale bars, 200 µm (zoom-in views of the indicated regions). **e** Quantitative analysis of infarct size in rats with myocardial infarction. **p < 0.005

To assess the level of maturity of CMs differentiated in transferrin-supplemented medium relative to those in B27-ins medium as well as primary cells in vivo human development, we compared a series of RNA-seq data, including three time points of our data (day 15, 21, and 35), three time points of hiPSC-derived CMs provided by Churko et al. (day 14, 30, and 90) (Churko et al. 2018), and human fetal (day 91 and day 120 embryo heart) and adult heart (atrium, left ventricle, and right ventricle) from ENCODE. The expression pattern of genes representing early fetal (*ATP2A2*, *MYH6*, and *TNNI1*) and late stages of heart development (*MYH7*, *MYL2*, and *TNNI3*) reflected similar results in CMs differentiated in transferrin-supplemented medium and B27 -ins medium, which closely resembled embryonic CMs but remained more immature than the adult heart (Fig. 3d).

Together, transcriptome-wide expression analysis suggested that CMs differentiated in the transferrin-supplemented medium were comparable to those commonly generated in B27-ins medium.

### Transplantation of hPSC-derived CMs improves cardiac function

**Fig. 5:**
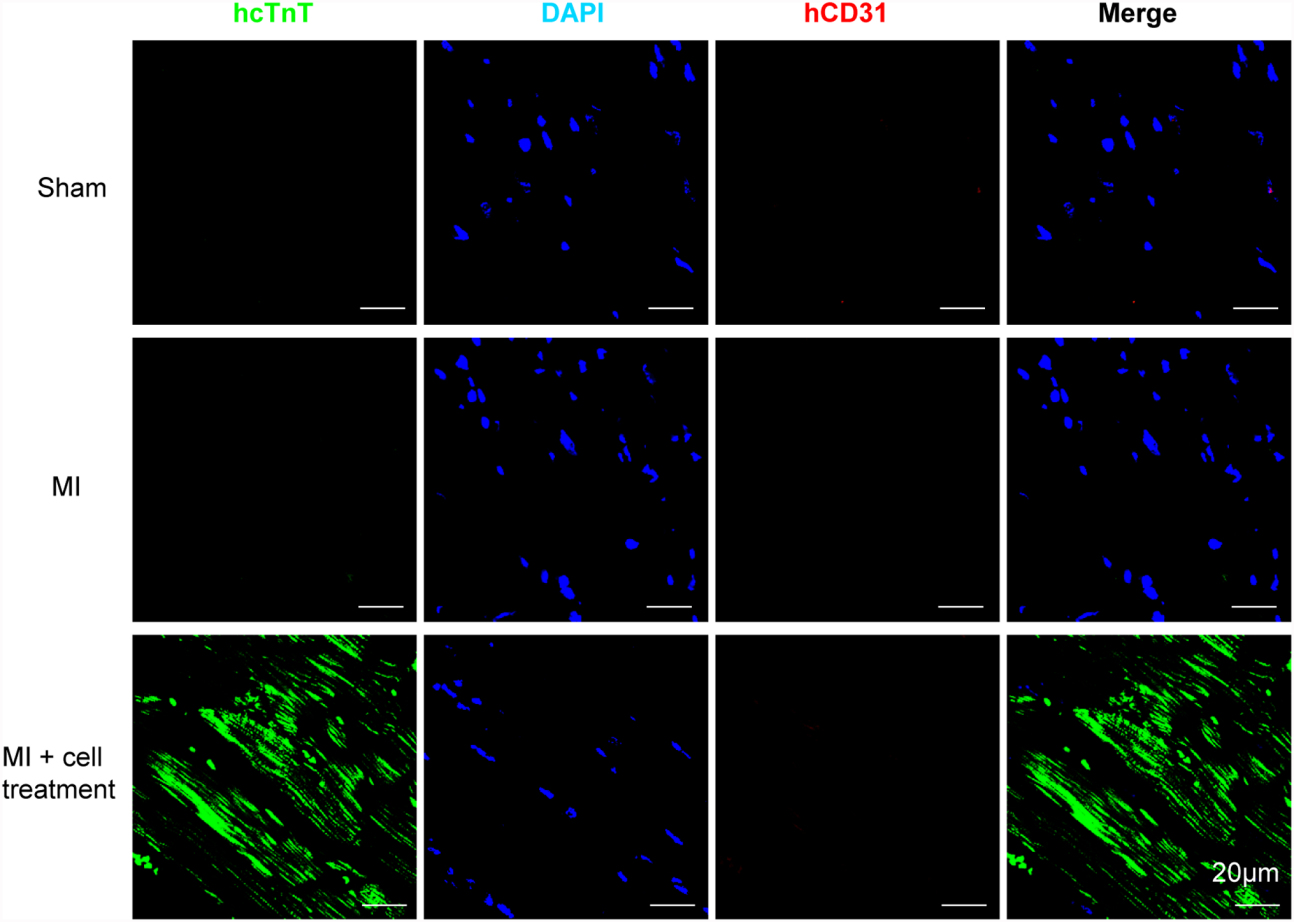
Remuscularization of the infarcted rat heart after hiPSC-derived CM transplantation. Representative confocal images of rat hearts stained for human cardiac troponin T (hcTnT, green) and human CD31 (hCD31, red) on day 28 post-transplantation. Scale bars, 20 µm

To evaluate the in vivo functionality of hPSC-derived CMs in our differentiation system, we delivered these cells to engraft to host myocardium in a rat model of myocardial infarction (MI). MI was created by permanent ligation of the left anterior descending coronary artery (LAD). After MI, we injected 8 × 10^6^ hiPSC-derived CMs into the infarct area and surrounding border zones. Echocardiographic measurements were performed 3 days and 28 days after cell transplantation and cardiac performance was assessed by two key parameters of left ventricle fractional shortening (LVFS) and left ventricle ejection fraction (LVEF). At 3 days after cell transplantation, MI rats showed significantly reduced LVFS and LVEF, but there was a moderate increase in LVFS and a significant increase in LVEF in MI rat hearts receiving hPSC-derived CMs (MI + cell treatment) (Supplementary Fig. 5). By day 28, there was a significant decrease of LVFS (18.59 ± 7.51%) and LVEF (38.84 ± 16.78%) in MI rat hearts compared to Sham group’s LVFS (39.31 ± 4.77%) and LVEF (70.79 ± 5.49%) (Fig. 4a–c). Transplantation of cells significantly increased LVFS (29.30 ± 5.71%) and LVEF (61.77 ± 9.06) (Fig. 4a–c). After echocardiography examination, rat hearts were harvested for histological analysis. Masson’s trichrome staining showed that MI + cell treatment group significantly reduced the fibrotic area by 16% compared with Control group (Fig. 4d and e), indicating less pathological remodeling of the myocardium. Staining with an antibody against human cTnT revealed the appearance of human myocardium within the infarct area (Fig. 5), suggesting better remuscularization of hiPSC-derived CMs engrafted hearts. Thus, CMs differentiated in the transferrin-supplemented medium are capable of contributing to the remuscularization of the infarcted heart in vivo.

## Discussion

In this study, we develop a manageable and efficient protocol for monolayer-based hPSC cardiac differentiation using a fully defined and cost-effective culture medium. The medium was prepared by adding just three supplements (recombinant human albumin, ascorbic acid, and human transferrin) into the basal medium RPMI 1640. Compared with the simplest CDM3 medium reported by Burridge et al. (Burridge et al. 2014), we found the addition of transferrin has two key roles: (1) Promoting hPSCs to smoothly switch and successfully start mesoderm induction from TeSR-E8 medium to rigorous cardiac differentiation medium; (2) Helping hPSCs go through cardiac differentiation even in the case that the blockage of Wnt signaling pathway caused high cell death rates during cardiac specification. This way, the protocol has much better reproducibility and little bias against operators. Our protocol produced CMs at the efficiency of approximately 85% cTnT^+^ cells similar to other methods previously reported. Transcriptome-wide expression signature reflects these CMs closely resemble those from B27-ins supplemented medium. Importantly, these hPSC-derived CMs could be used to remuscularize human myocardium in the infarcted heart in vivo.

The minimal system for cardiac differentiation holds great potential for large scale cell production and application. In fact, one of the biggest obstacles is cell death during cardiac differentiation when we used CDM3 medium just including RPMI 1640, ascorbic acid, and human albumin (Burridge et al. 2014). Given that high levels of insulin and transferrin in TeSR-E8, hPSCs suffer significant cell death in sudden withdrawal of them during differentiation. Here, we speculated that transferrin was required for cell survival and smooth transition when hPSCs initiated cardiac differentiation in the absence of insulin. If it were completely removed, cells would be more vulnerable and tend to vary due to external culture environment or operation action. As expected, we demonstrated that transferrin was crucial for CM differentiation, except for albumin and ascorbic acid. It could support hPSCs going through cardiac differentiation and was more resistant to the changes of some uncontrollable environmental factors. Transferrin is widely recognized as an iron-binding protein and plays an important role in multiple biological actions, such as cell growth and cytoprotective activities (Gomme and McCann 2005; Wei et al. 2015). The utility of transferrin in E8 medium for pluripotent cell culture and in B27-ins for cell differentiation suggests the fundamental roles in cell life activities. Selenium (Se) is also well known for its antioxidant activity in cell survival and cardioprotection (Dursun et al. 2011; Liu et al. 2013; Schmidt et al. 2018). When we added sodium selenite into the culture medium, there was little influence on improving cell survival or cardiac differentiation efficiency (Supplementary Fig. 3), indicating the priority of albumin, ascorbic acid, and transferrin.

With the transferrin-supplemented AA medium for the cardiac differentiation, all tested cell lines, including three hESC lines (H1, h9, and HUES8) and one hiPSC line (CD34-iPSC), were not subject to total cell death any more unless mycoplasma contamination occurred. Cell density and the concentration of CHIR99021 treatment were the two main optimizable factors for successful differentiation. First of all, the starting cell density is ideally controlled by achieving 80–90% confluence in 3–4 days, which is a precondition for a high success rate. For this reason, the split ratio of hPSCs should be adjusted flexibly according to the proliferation rates of different cell lines. Next, the suitable concentration of CHIR99021 treatment varies among different hPSC cell lines due to the cell context in response to small molecules (Cao et al. 2013; Burridge et al. 2015). For example, we found the dose of CHIR99021 strictly limited at the range of 3–5 μM in our tested hPSC lines, which is essential to the efficiency of the protocol. For the same cell line, as appropriate, 1 μM of CHIR99021 might fluctuate up and down for early-passage lines (less than 30). In order to test the operability and stability of this protocol, differentiation experiments were carried out by 5 beginning graduate students without targeted training in different laboratories. Excitingly, they all differentiated beating CMs following the prepared protocol within 3 times. Together, the transferrin-supplemented AA medium makes CM differentiation even easier to handle just following these two points above.

The resultant CMs showed considerable efficiency, purity, as well as typical phenotypic properties. In the details of the molecular signature, genome-wide expression analysis revealed a temporal expression pattern of stage-specific genes navigating cardiac lineage commitment during the cardiac differentiation process, reflecting the consistency with developmental trajectories in vivo. In our differentiation system, hPSCs begin with a high level of pluripotency marker (*DNMT3*, *NANOG*, and *POU5F1*) at day 0, and transit through mesoderm (*MESP1* and *MIXL1*) at day 2 into cardiac progenitor (*ISL1* and *HAND1*) at day 5, committed (*TNNI1*) at day 15 and definitive cardiac state (*MYH7*) at day 35, keeping in step with the protocol described previously (Palpant et al. 2016). Interestingly, Spearman correlation coefficients and PCA on the time-course RNA-seq data revealed hPSCs underwent dramatic changes in gene expression patterns as mesoderm induction and subsequent cardiac specification was directed by manipulation of Wnt signaling in the first 4 days, and only modest differences appeared until the cardiac fate committed after differentiation day 15. To further define the identity and maturity of the hPSC-derived CMs, we compared our committed and definitive CMs (including three points of day 15, 21, and 35) against reference RNA-seq data such as CMs (day 14, 30, and 90) generated in B27-supplemented medium, and data from fetal and adult hearts (ENCODE) (Churko et al. 2018; Friedman et al. 2018). Using genes that represent either early stage (*ATP2A2*, *MYH6*, *TNNI1*, *NKX2-5*, and *GATA4*) versus late stage of heart development (*MYH7*, *MYBPC3*, *ACTN2*, *TNNI3*, and *MYL2*) (Piccini et al. 2015; Churko et al. 2018; Friedman et al. 2018), expectedly, all hPSC-derived CMs are quite similar to each other and very close to their embryonic counterparts rather than adult heart, indicating the most differentiated hPSC-derived CMs (even more than 30 days in vitro culture) still need advanced maturation by electrical, mechanical, and hydrodynamic stimulation (Shadrin et al. 2017; Tiburcy et al. 2017; Ronaldson-Bouchard et al. 2018). Thus, long-term culture in dish alone is not enough to develop the feature of adult myocardium, which forces us to explore more powerful strategies for cardiomyocyte maturation in the following studies.

And more importantly, transplanted hPSC-derived CMs could remuscularize damaged myocardium tissue and led to significantly improved physiological function in a rat model of MI, indicating the clear functionality in vivo and potential application in regenerative strategies. Although progress has been made in improving cardiac performance in animal MI models (rodents, swine, and nonhuman primates) by transplanting hPSC-derived CMs, such as long-term survival of grafts and contractile function in synchrony with host myocardium (Chong et al. 2014; Shiba et al. 2016; Liu et al. 2018), optimal cell production in fully chemically defined system is quite beneficial for the translation of hPSC-derived CMs to clinical applications. Furthermore, cell delivery is a critical link in cell transplantation and is greatly supported by tissue engineering technologies for recent years. Cardiomyocyte development is intimately connected with the surrounded other cardiac cell types, therefore, in the future, an attractive option could be a multipronged strategy to develop 3D myocardial constructs with extracellular matrix microenvironment supported cell communication, which facilitates cardiomyocyte development and transplantation approaches, ultimately achieving better cardiac repair (Ogle et al. 2016; Richards et al. 2017).

## Conclusions

In summary, the transferrin-supported cardiac differentiation system ensured an ease-of-use and cost-effective platform for hPSC-derived CM generation. Transferrin was necessary for hPSCs getting through monolayer-based cardiac differentiation and successfully developing into target cells. We extensively described their identity, efficiency, maturity, as well as in vivo remuscularization function. Our optimization maximizes the reproducibility of cardiac differentiation free from some uncontrollable environmental factors and facilitates the widespread application of hPSC-derived CMs for heart repair.

## Electronic supplementary material

Below is the link to the electronic supplementary material.

Electronic supplementary material 1 (PDF 1291 kb)

Electronic supplementary material 2 (MP4 12631 kb)

Electronic supplementary material 3 (MOV 15345 kb)

Electronic supplementary material 4 (DOCX 17 kb)

## Data Availability

RNA-seq data are publicly at the Gene Expression Omnibus (GEO) with accession number GSE154294. All other relevant data and materials are available within the paper and its supplementary information files.

## Code availability

This study used commercial and open-source software packages, which are specified in the Materials and methods.
