# Supplementary material for: Transferrin improved the generation of cardiomyocyte from human pluripotent stem cells for myocardial infarction repair
Source: J Mol Histol. 2020 Nov 11;52(1):87–99. doi: 10.1007/s10735-020-09926-0 (PMC7790792; doi:10.1007/s10735-020-09926-0)

## Supplementary Fig. 1 Optimization of transferrin dose for CM differentiation

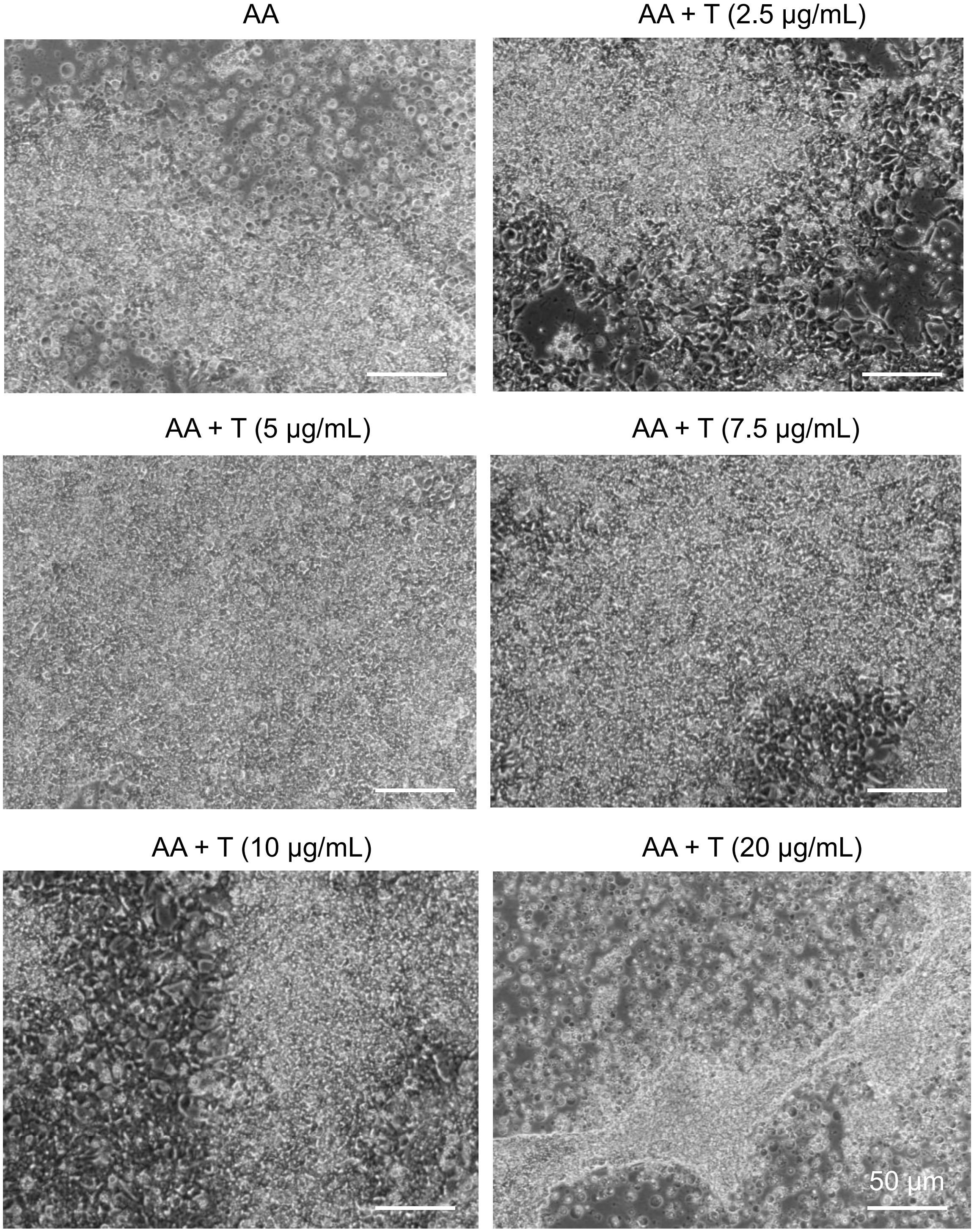

**Supplementary Fig. 2 Simultaneous confocal imaging of $Ca^{2+}$ transient in day 21 hiPSC-CMs**

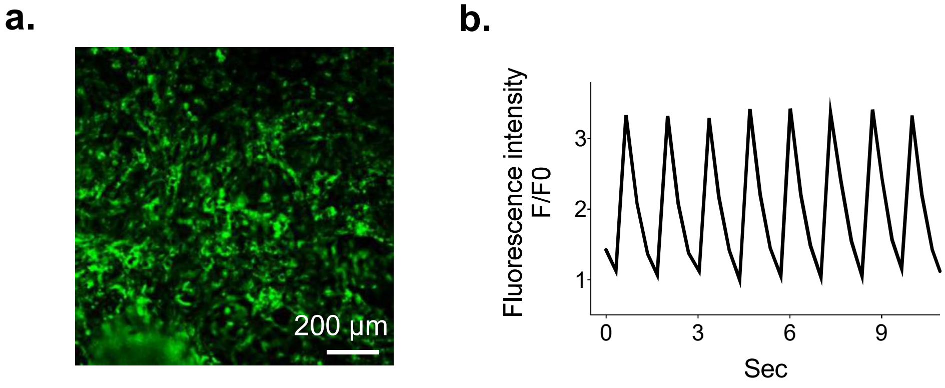

**Supplementary Fig. 3 Assessment of addition of sodium selenite on cardiac differentiation**

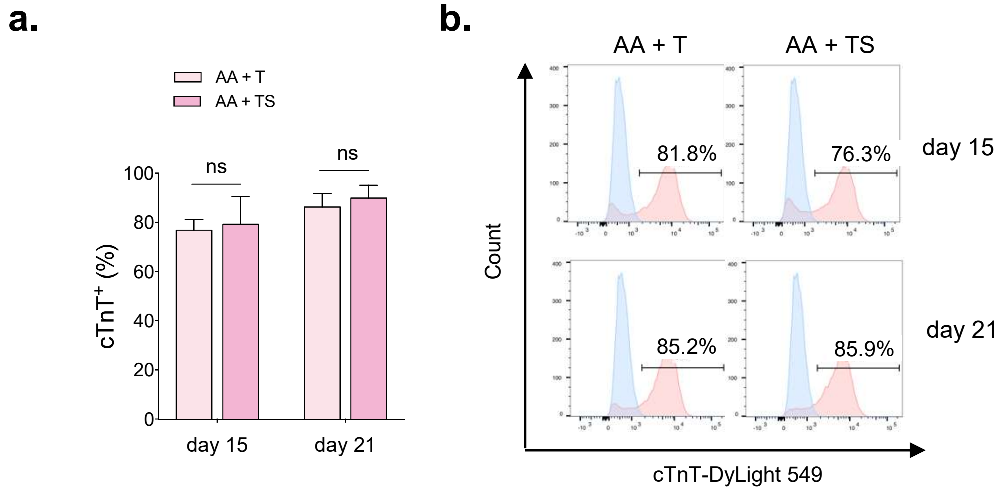

# Supplementary Fig. 4 Q-PCR validation of stage-specific genes during hiPSC-derived cardiac differentiation according to the RNA-seq results

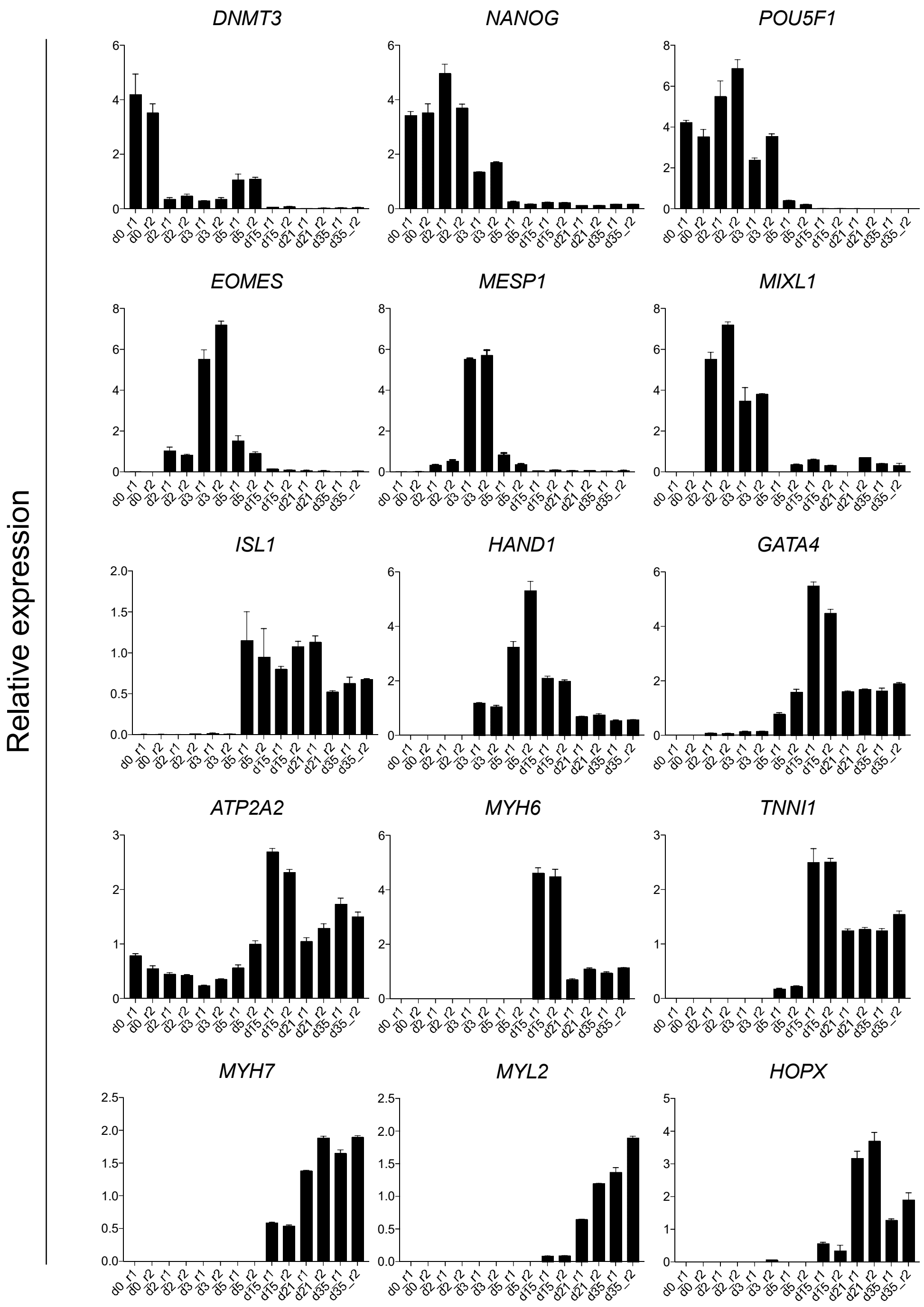

**Supplementary Fig. 5** **Quantitative cardiac function analysis of LVFS and LVEF on day 3 post-transplantation**

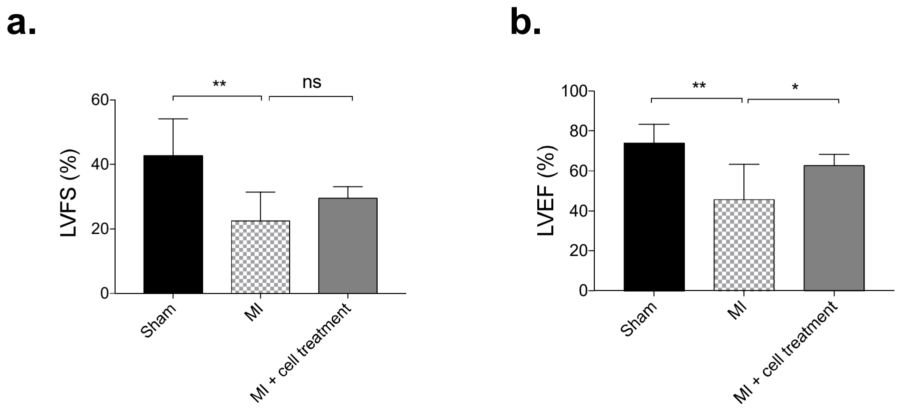

Supplement: Supplementary file 1 — Electronic supplementary material 1 (PDF 1291 kb) [file 10735_2020_9926_MOESM1_ESM.pdf]
