# Supplementary material for: Transferrin improved the generation of cardiomyocyte from human pluripotent stem cells for myocardial infarction repair
Source: J Mol Histol. 2020 Nov 11;52(1):87–99. doi: 10.1007/s10735-020-09926-0 (PMC7790792; doi:10.1007/s10735-020-09926-0)
Supplement: Supplementary file 4 — Electronic supplementary material 4 (DOCX 17 kb) [file 10735_2020_9926_MOESM4_ESM.docx]

Journal of Molecular Histology

**Supplementary Information**

**Transferrin improved the generation of cardiomyocyte from human pluripotent stem cells for myocardial infarction repair**

Fengzhi Zhang^1^, Hui Qiu^2^, Xiaohui Dong^1^, Chunlan Wang^1^, Jie Na^2^, Jin Zhou^1^, and Changyong Wang^1, *^

^1^Department of Neural Engineering and Biological Interdisciplinary Studies, Institute of Military Cognition and Brain Sciences, Academy of Military Medical Sciences, Academy of Military Sciences, Beijing, China

^2^School of Medicine, Tsinghua University, Beijing, China

^*^Correspondence: Changyong Wang^1^, E-mail: [wcy2000_zm@163.com](mailto:wcy2000_zm@163.com)

**Supplementary Tables**

**Table 1. Primary Antibodies**

| Antigen | Host | Dilution | Vendor | Catalog Number |
| --- | --- | --- | --- | --- |
| cTnT | mouse | 1:200 | R&D Systems | AF938 |
| ⍺-actinin | mouse | 1:200 | R&D Systems | BBA3 |
| MLC2a | mouse | 1:200 | Synaptic Systems | 311011 |
| MLC2v | rabbit | 1:200 | Proteintech | 10906-AP |
| hCD31 | rabbit | 1:200 | Abcam | ab32457 |
| hcTnT | goat | 1:200 | Abcam | ab64623 |

**Table 2. Secondary Antibodies**

| Antigen | Host | Dilution | Vendor | Catalog Number |
| --- | --- | --- | --- | --- |
| anti-rabbit IgG DyLight 488 | goat | 1:500 | Thermo | 35552 |
| anti-mouse IgG DyLight 550 | goat | 1:500 | Thermo | 84540 |
| anti-goat IgG Alexa Fluor 488 | Donkey | 1:500 | Lifetechnology | A-11055 |
| anti-mouse IgG DyLight 488 | goat | 1:500 | Thermo | 35502 |
| anti-rabbit IgG DyLight 550 | goat | 1:500 | Thermo | 84541 |

**Table 3. Primers for Q-PCR**

| Gene | Forward primer | Reverse primer | T_m_ |
| --- | --- | --- | --- |
| *GAPDH* | ﻿tgatgacatcaagaaggtggtgaag | ﻿tccttggaggccatgtgggccat | 60 |
| *DNMT3* | ggaaattagaatcaaggaaatacga | aatttgtcttgaggcgcttg | 60 |
| *POU5F1* | tgagtagtcccttcgcaagc | gagaaggcgaaatccgaag | 60 |
| *NANOG* | ggatggtctcgatctcctga | cctcccaatcccaaacaata | 60 |
| *EOMES* | accttcttccagcgtgtgag | tcctcgtacctcttgctcct | 60 |
| *MESP1* | ﻿cgctgtgccccgacgact | ﻿ggcatccaggtctccaacag | 60 |
| *MIXL1* | ggtaccccgacatccactt | cctggaagaggggagaaaat | 60 |
| *ISL1* | ﻿ctgcacaccttgcggacctg | ﻿caccgtcgtgtctctctggac | 60 |
| *HAND1* | aactcaagaaggcggatgg | ggaggaaaaccttcgtgct | 60 |
| *GATA4* | ggaagcccaagaacctgaat | gttgctggagttgctggaa | 60 |
| *ATP2A2* | aacgtcggggaagttgtct | gaatcaaagcctcgggaaat | 60 |
| *MYH6* | tgctcagagctcaagaaggac | cccagccatctcctctgtta | 60 |
| *TNNI1* | gggccaacctcaagtctgt | agacatggcctccacgtt | 60 |
| *MYH7* | acaccctgactaaggccaaa | gtccatgcgcaccttctt | 60 |
| *MYL2* | gcaggcggagaggttttc | agttgccagtcacgtcagg | 60 |
| *HOPX* | cagcaaacacagcttccaaa | tgctccgctagacccttct | 60 |
